# Anthropometrics, Performance, and Psychological Outcomes in Mixed Martial Arts Athletes

**DOI:** 10.3390/biology11081147

**Published:** 2022-07-30

**Authors:** Nathalia Ferreira Camarco, Ivo Vieira de Sousa Neto, Evaldo Ribeiro, Anderson J. Martino Andrade

**Affiliations:** 1 Department of Physiology, University of Parana, Curitiba 81531-990, Brazil; martino.andrade@gmail.com; 2 Laboratory of Molecular Analysis, Faculty of Ceilândia, Universidade de Brasília, Brasília 70910-900, Brazil; ivoneto04@hotmail.com; 3 Department of Physical Education, University of Parana, Matinhos 83260-000, Brazil; evaldo.ribeirojr@ufpr.br

**Keywords:** combat sports, digit ratio, handgrip strength, aggressiveness, personality

## Abstract

**Simple Summary:**

The digit ratio is a prenatal testosterone biomarker and it is related to sports performance. Few investigations have explored the digit ratio in determining the potential performance of an individual in power-based sports, specifically, combat sports. This biomarker, in conjunction with other measures, might be valuable in determining the potential athleticism of an individual. In the present study, we compared the digit ratios between mixed martial arts athletes and non-athletes. Moreover, we investigated the association between (1) handgrip strength and the digit ratio in mixed martial arts athletes and (2) psychometric variables and digit ratios in mixed martial arts athletes and non-athletes. Athletes presented lower digit ratios than the control group in both hands. Moreover, athletes presented less agreeableness, more openness, and less anger than the controls. We observed that the left hand 2D:4D was negatively correlated with handgrip strength and lean body mass in professional athletes. However, we did not observe an association between psychometric variables or wins with 2D:4D. To conclude, the prior digit ratio might provide efficient data for analyses and important feedback to determine key performance predictors in a particular competition, but it does not explain the full sports performance; it needs to be included with other performance measures.

**Abstract:**

The digit ratio (2D:4D) is related to prenatal testosterone (T) and sports performance. Few investigations have explored 2D:4D in determining the potential performance of individuals in power-based sports, specifically combat sports. This study compared 2D:4D between mixed martial arts (MMA) athletes and non-athletes and investigated the association between (1) handgrip strength (HGS) and lean body mass (LBM) with 2D:4D in MMA athletes and (2) psychometric variables and 2D:4D in MMA athletes and non-athletes. In total, 122 men participated in this study (53 non-athletes, 45 professionals, and 24 amateur fighters). The 2D:4D was measured using a caliper, HGS was assessed with a dynamometer, and psychometric variables were evaluated using questionnaires. Athletes displayed significantly (*p* < 0.05) lower median values of right (0.95 ± 0.04) and left (0.96 ± 0.03) 2D:4D in comparison with non-athletes, (0.97 ± 0.03) and (0.99 ± 0.03), respectively. We observed that left hand 2D:4D was negatively correlated with HGS (r = −0.43; *p* < 0.05) and lean body mass (r = −0.49; *p* < 0.05) in professional athletes. Professional athletes also displayed significant differences (* *p* < 0.05; ** *p* < 0.001) in psychometric variables compared to non-athletes for (1) agreeableness **, median values (interquartile range) = −0.11 (−0.19–0.07) and 0.2 (−0.09–0.33), respectively; (2) anger **, mean ± standard error = 2.40 ± 0.12 and 2.89 ± 0.89, respectively; and (3) openness *, mean ± standard error = 0.17 ± 0.04 and −0.006 ± 0.04, respectively. However, we did not observe an association between psychometric variables or wins with 2D:4D. In conjunction with other measures, 2D:4D is valuable in determining the potential athleticism of an MMA athlete.

## 1. Introduction

Mixed martial arts (MMA) is a full-contact sport that combines a range of martial arts techniques and various combat sports [1]. There is evidence that there is a range of genetic and epigenetic factors that contribute to successful performance in sports, such as MMA, including training routines, diets, recovery, psychological threats, and environmental aspects [2]; for theoretical reasons, this may also include the in utero environment.

The digit ratio (2D:4D) is calculated using the ratio of the second to the fourth fingers of the hands, and it is inversely related to prenatal testosterone (T) and directly related to prenatal estrogen. It is usually defined as a sexually dimorphic biomarker because cisgender males tend to display lower values than cisgender females [3,4]. Research shows a lack of an association between 2D:4D and basal testosterone levels in adults [5,6]; however, it is known that the in utero concentrations of sex hormones may have permanent developmental effects on the brain and cardiovascular morphology (i.e., organizational effects), which may explain why 2D:4D has been described as a potential indicator of sports success [7].

It has been reported that 2D:4D strongly predicts endurance performance [8], individual variations in maximal oxygen uptake (VO_2max_), and high maximum lactate concentrations in teenage boys [9]. Furthermore, several studies associated 2D:4D with sports performances [10], mainly in racers [8] and soccer players [7], but few investigations have explored the value of 2D:4D in determining the potential performance of an individual in power-based sports, specifically, combat sports [11]. Currently, there is evidence of associations between lower 2D:4D and (1) female [12] and male [11] choices of combat sports, and (2) the percentage of wins in male sumo wrestlers [13]. Furthermore, lower values of 2D:4D have been reported in male elite wrestler athletes compared with amateurs [14].

In a systematic review designed to assess the determinants of sports performance in MMA athletes [15], it has been reported that anaerobic rather than aerobic qualities are the main indicators that distinguish the higher and lower-level combat sports athletes. A well-prepared MMA athlete will need to possess high levels of maximal strength and strength endurance accompanied by the ability to express power (repeatedly) under loaded conditions [16]. Based on these findings, handgrip strength (HGS) might be an interesting putative biomarker used to measure the athletic performances of MMA athletes. A strong grip is particularly important for athletes to dominate their opponents during grappling tie-ups encountered in a fighting competition [17]. However, the association between 2D:4D with HGS in MMA athletes has not yet been investigated. A relationship between 2D:4D and HGS appears plausible because in utero male sexual differentiation and gonadal development depend on T release [18]; higher levels of T may exert effects of increased muscle strength [19].

In addition to the physiological explanation, the effects of psychology on sports performance have been long recognized. The association between physical fitness and low 2D:4D might be mediated through an association with exercise frequency, suggesting that the organizational effects of prenatal T may predict a type of behavior that interferes with exercise frequency and leads to increased strength and performance in male athletes [20]. This could lead to possible psychological differences between combat sports athletes and non-athletes (as important features of competitors).

In this context, shedding light on physiological or psychometric variables that might be associated with 2D:4D may guide coaches to adjust individualized training approach strategies, to be able to timely intervene [21]. Prior 2D:4D, HGS, and screening for physiological factors might provide efficient data for analyses and important feedback to determine key performance predictors in a particular competition. This information could be valuable for evidence-based strategies during competitions and to identify possible deficient athletic performances. Thus, the current study compared the digit ratios (2D:4D) between MMA athletes and non-athletes and investigated the association between (1) muscular strength/lean body mass (LBM) and 2D:4D in MMA athletes and (2) psychometric variables and 2D:4D in MMA athletes and non-athletes. We also evaluated the association between 2D:4D and win outcome. We hypothesize that athletes will display lower values of 2D:4D and that HGS may be negatively related to prenatal androgenization. In addition, it is expected that psychological traits that may lead to high performances, such as conscientiousness and openness, will differ in athletes in comparison with non-athletes.

## 2. Materials and Methods

### 2.1. Participants

**Figure 1:**
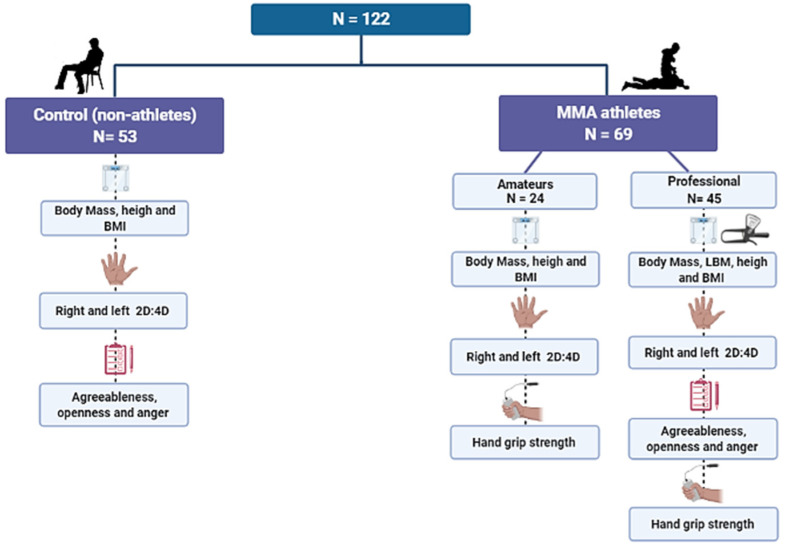
Sample distribution and dependent variables.

In total, 122 men participated in this study. A total of 69 MMA athletes, of which 45 professional athletes, defined as MMA athletes with at least two professional fights, and 24 amateur athletes, were recruited from 9 fighting academies in Curitiba, Brazil. Additional inclusion criteria for participating athletes included being free of osteoarticular lesions that interfered with the HGS test and not using anabolic steroids or dietary supplements, which provide strength gains. Participants who used creatine up until 1 month before data collection were excluded from the HGS test. Furthermore, 53 college students between 18 and 40 years old, were recruited, following the same additional inclusion criteria (Figure 1).

Data for sample size calculations were obtained from a previous study that used 2D:4D in combat athletes [11]. A minimum sample size of 46 participants per group was estimated based on the ability to detect a minimal meaningful difference in the 2D:4D analysis, considering a type I error level (α) of 0.05 and a power of 80% to detect a large effect (ηρ2 > 0.60). Calculation of the sample size was performed using G*Power Software (version 3.0.10, Germany) [22].

### 2.2. Experimental Design

All athletes separately visited the laboratory at 9:00 am on the scheduled day, in a fasting state, to complete the experimental protocol. At first, the body density was estimated through an equation including the sum of the mean (in triplicate) of seven skinfold measurement sites (triceps, subscapular, pectoral, midaxillary, supra-iliac, abdominal, and medial femoral), conducted by a single researcher using a calibrated Lange caliper (Beta Technology, Santa Cruz, CA, USA). Body density was used to calculate fat mass. LMB was calculated as total body mass–fat mass. [23,24]. Next, the athletes provided their ages, body masses, and heights, and completed two self-administered psychometric questionnaires—the big five inventory (BFI), designed to measure five personality traits, and the Buss–Perry aggression questionnaire (BPAQ), a 26-item questionnaire designed to assess aggression in four dimensions. Following the questionnaires, the 2D:4D digit ratio and the handgrip strength were measured. BMI was calculated by the equation: body mass/(height)^2^ [25]

The students took part in a shorter protocol, which consisted of the same BFI and BPAQ questionnaires administered to the athletes, followed by the assessment of the 2D:4D digit ratio, measurement of body mass, and information on self-reported height. The questionnaires and 2D:4D measurements were performed in the students’ classrooms after signing the informed consent. Each participant received the questionnaire separately and was instructed to remain silent while responding.

Participants were fully informed of any risks and discomforts associated with the experiments before giving their informed written consent to participate. The study was approved by the Institutional Review Board (IRB) of the Federal University of Paraná (n. 91580318.6.0000.0102). After recruitment and signing the informed consent terms, the athletes were encouraged to refrain from all dietary sources of caffeine, alcohol, or stimulants for 24 h before the experimental trial for HGS.

### 2.3. Digit Ratio (2D:4D) Assessment

Direct measurements of the 2nd (index) and 4th (ring) fingers from the mid-point on the ventral crease proximal to the palm to the tip of the finger were taken twice. A digital caliper with 0.01 mm precision (Digimatic Caliper, 500–196 series, Mitutoyo Corporation, Sakado, Japan) was used. The complete protocol is described by [3]. The measurements were taken from both left and right hands. The 2D:4D of both hands showed high and significant ICCs for the first and second measurements (right 2D:4D, ICC = 0.98, F = 147.92, *p* < 0.0001; left 2D:4D, ICC = 0.99, F = 244.89, *p* < 0.0001). Thus, we used the means for left and right 2D:4D in all subsequent tests [5].

### 2.4. Hand Grip Strength (HGS)

At first, a study examiner demonstrated how to perform the test. The participants then performed a simulation of a test performing submaximal strength for familiarization with the equipment. After one minute of rest, the main test was performed. Participants were instructed not to look at the dynamometer dial (to avoid any visual feedback). Instructions for the test executions were standardized by an experienced evaluator. During the instructions, the volume of the verbal command remained constant to avoid any influence on the magnitude of muscle contraction [26].

Participants stood with the superior limbs extended to the side of the body; the medial phalanges of the fingers placed pressure on the shaft of the device [27]. They performed handgrip maximal force production tests in both hands measured with a manual handgrip dynamometer (Grip-D, Takei, Japan). Participants conducted 2 attempts with each hand, with 1 min of recovery between repetitions; the highest value was used for the analysis [28].

Although the handgrip strength is typically greater on the dominant side, the differences between sides vary widely among studies and depend on whether individuals are right- or left-hand dominant. The available information may be insufficient to justify using between-side comparisons to make judgments about grip strength [29]. Considering the specific grappling bilaterality of the sport, we included the maximum HGS obtained in the test for the analysis. To control the influence of body mass on HGS, we used the allometric scaling of body mass, following the equation: F_nor_ = F/m^b^, where F_nor_ represents the allometric adjusted HGS (or HGS index), F represents absolute muscle strength, m represents the body mass in kg, and b is the allometric exponent (beta coefficient) obtained from the linear regression; the natural logarithm of body mass is an independent variable and the natural logarithm of maximum HGS is a dependent variable [30].

### 2.5. Questionnaires

#### 2.5.1. Big Five Inventory (BFI)

The Portuguese validated [31] BFI personality model is hierarchical, with scores from 1 to 5, on a Likert scale model, grouped into 5 broad dimensions: (1) Openness—refers to exploratory behaviors. (2) Conscientiousness, lack of impulsivity, or will. In this factor, impulse controls are characteristic, as well as behaviors directed toward specific objectives. (3) Extroversion—the component of human personality; in the big five factor model, this is related to how people interact with others. (4) Agreeableness—characterizes orientations toward others, including traits such as altruism, trust, and modesty. (5) Neuroticism—contrasts emotional instability with negative effects, including anxiety, sadness, irritability, and nervous tension.

We calculated the ipsatized index for each factor. Ipsatization, or intra-subject standardization, is a method that recodifies the raw scores based on the mean and standard deviations of some positive and negative items (response pairs) of each participant [32]. We also performed the acquiescence index. Acquiescence refers to the tendency to respond positively to items, despite their descriptive content. To try to eliminate this effect, questions about the same factors were presented, alternating between positive and negative [33].

#### 2.5.2. Buss–Perry Aggression Questionnaire (BPAQ)

Aggressiveness was assessed using the aggressiveness questionnaire (BPAQ) (Buss & Perry, 1992). This instrument is based on the Buss–Durkee hostility inventory (BDHI), developed by [34]. The validated BPAQ version in Portuguese [35] consists of 26 items and assesses aggression in four dimensions: physical aggression (8 items), verbal aggression (4 items), anger (6 items), and hostility (8 items). Scores were measured on a 1 to 5 Likert scale and the mean value was calculated for each dimension [36].

### 2.6. Statistical Analysis

At first, we used the Shapiro–Wilk test to assess the normality in groups of athletes and non-athletes. Differences between groups were analyzed using a non-paired T-test for parametric variables and a Mann–Whitney U test for non-parametric variables. We performed Spearman’s correlation between 2D:4D and HGS in athletes. Spearman’s correlation was also performed between 2D:4D and BFI agreeableness, BFI openness, and BPQ anger in both groups. We classified results for the correlation index as: 0.00–0.19 very weak, 0.20–0.39 weak, 0.40–0.69 moderate, 0.7–0.89 strong, 0.9–1.0 very strong [37].

Moreover, the chi-square test was used for the association between prenatal androgenization and the winning outcome. For this comparison, participants were divided into two groups (high and low) of 2D:4D and two groups (high and low) of frequency of wins, according to the 50th percentile. The high 2D:4D was defined as ≥0.94 for the right hand and ≥0.96 for the left hand. The high winning frequency was defined as ≥0.68. No clinical or physiological criteria have been established in the literature to define the cutoff value of the percentile. Hence, initially, we performed an exploratory analysis to determine the optimal contrast between groups. Stratifying into halves displayed a stronger contrast between groups when compared to thirds or fourths. The level of significance was set at *p* < 0.05. Data were analyzed using IBM SPSS Statistics, version 26, for Mac.

## 3. Results

### 3.1. Descriptive Statistics and Comparison of Digit Ratios

**Figure 2:**
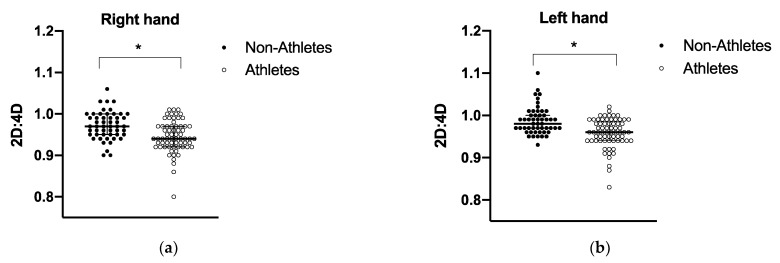
Median and interquartile ranges for right (**a**) and left (**b**) 2D:4D ratios in athletes and non-athletes. Mann–Whitney U test. Results are described in median and interquartile ranges. Athletes, n = 66; non-athletes, n = 51. * *p* > 0.0001.

Descriptive statistics are shown in Table 1. The sample consisted of 69 MMA athletes and 53 college-aged students. The median age (y) of the athlete’s group was slightly higher, i.e., 25 (18–39) than the non-athletes, i.e., 22 (18–35). Weight, height, and BMI did not differ between groups (Figure 2). As expected, digit ratios were significantly different between groups, in both hands, with athletes displaying more androgenized profiles, with lower median values of the right and left 2D:4D ratios, (0.95 ± 0.04) and (0.96 ± 0.03), respectively, compared with non-athletes, (0.97 ± 0.03) and (0.99 ± 0.03), respectively. No differences were found between amateur and professional athletes in the left or right 2D:4D ratio (data not shown).

### 3.2. Correlation between 2D:4D, LBM, and HGS

**Figure 3:**
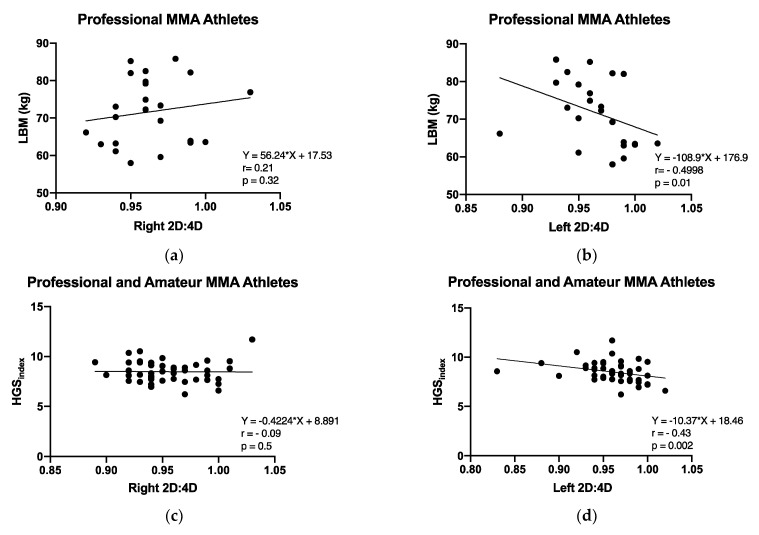
(**a**) Spearman’s correlation test between LBM and right 2D:4D among professional MMA athletes (*p* = 0.32. (**b**) Spearman’s correlation test between LBM and left 2D:4D among professional MMA athletes (*p* = 0.01). (**c**) Spearman’s correlation test between the HGS index and right 2D:4D among professional and amateur MMA athletes (*p* = 0.5). (**d**) Spearman’s correlation test between the HGS index and left 2D:4D among professional and amateur MMA athletes (*p* = 0.002).

After verifying normality in the HGS index data, we performed a T-test and verified that there were no differences between the HGS index in professional (n = 27) and amateur (n = 21) athletes (*p* = 0.575). Therefore, we tested the correlation between 2D:4D and the HGS index in all athletes (n = 48) for which we had both HGS and digit ratio measures. We found a moderate, negative, and significant correlation (r = −0.43, *p* = 0.002) between the left 2D:4D and the HGS index. No significant correlations were found with the right hand, (r = −0.09; *p* = 0.5; n = 48). Furthermore, we tested the correlation between 2D:4D and LBM in professional athletes, for which we had both LBM and digit ratio measures (n = 23). We found a negative significant correlation between the left 2D:4D and LBM (r = −0.499; *p* = 0.001). However, the right 2D:4D was not correlated with LMB (r = −0.21; *p* = 0.32; Figure 3).

### 3.3. Personality Traits in Professional MMA Athletes and Students

**Figure 4:**
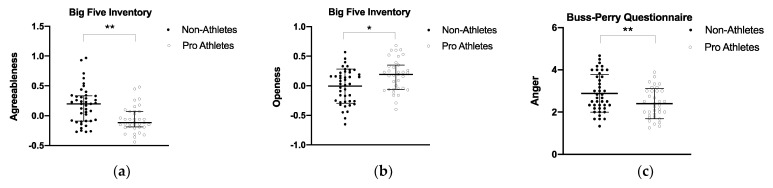
(**a**) Median and interquartile ranges for BFI agreeableness between groups of professional athletes (n = 35) and non-athletes (n = 42). (**b**) Mean and standard error for BFI openness between groups of professional athletes (n = 35) and non-athletes (n = 42). (**c**) Mean and standard error for BPQ anger between groups of professional athletes (n = 35) and non-athletes (n = 42). * *p* > 0.05 ** *p* > 0.001.

We found some differences in personality traits between groups. Results are described as the median and interquartile ranges for non-parametric data and the mean and standard error for parametric data (Figure 4). Athletes presented significantly lower ipsatized scores for BFI agreeableness than non-athletes, with median values (interquartile range) of −0.11 (−0.19–0.07) and 0.2 (−0.09–0.33), respectively. Moreover, athletes presented higher BFI openness scores (mean ± standard error = 0.17 ± 0.04) than non-athletes (−0.006 ± 0.04) and lower levels of aggression–anger: (mean ± standard error = 2.40 ± 0.12) and (2.89 ± 0.89) for athletes and non-athletes, respectively.

### 3.4. Correlation between 2D:4D and Agreeableness, Openness, and Anger

**Figure 5:**
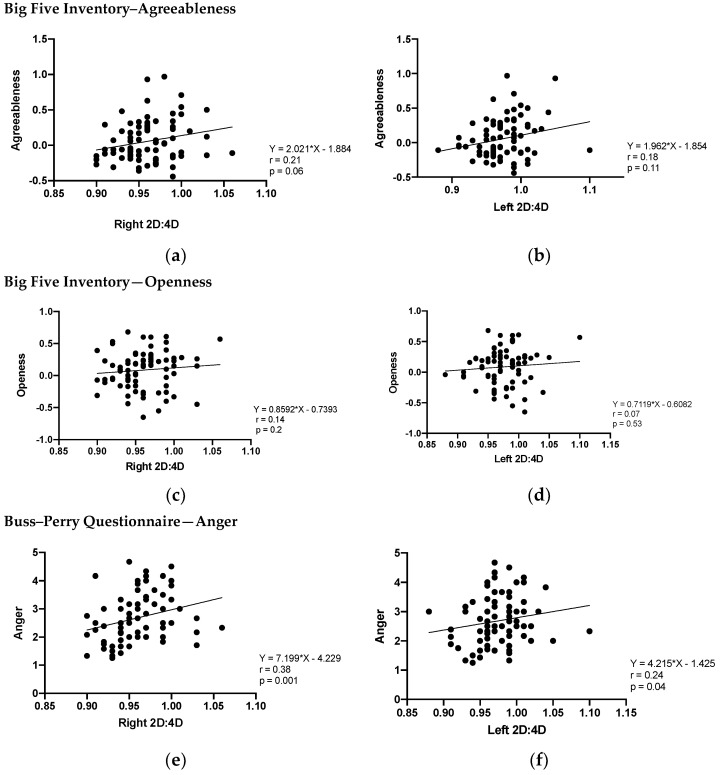
Spearman’s correlation between right 2D:4D and BFI agreeableness (**a**,**b**); BFI openness (**c**,**d**) and BPQ anger (**e**,**f**) in professional MMA athletes + non-athletes (n = 77). Significant differences (*p* > 0.05) only in BPQ anger for 2D:4D in both hands.

Considering the differences found in 2D:4D, BFI agreeableness, BFI openness, and BPQ anger between groups of professional athletes and non-athletes, we investigated the correlation of 2D:4D and BFI agreeableness, BFI openness, and BPQ anger in both groups together (professional athletes + non-athletes). We did not find any significant correlations between right or left 2D:4D and agreeableness and openness, except for a tendency in BFI agreeableness and right 2D:4D. On the other hand, anger was significantly correlated with both 2D:4D hands (Figure 5).

### 3.5. Association between Winning Outcome and 2D:4D

On the right hand, there was no statistically significant association between prenatal androgenization and win outcome (χ2(1) = 0.885, *p* = 0.346; Table 2). Similarly, on the left hand, there was no statistically significant association between prenatal androgenization and the winning outcome (χ2(1) = 0.277, *p* = 0.598; Table 2).

## 4. Discussion

**Figure 6:**
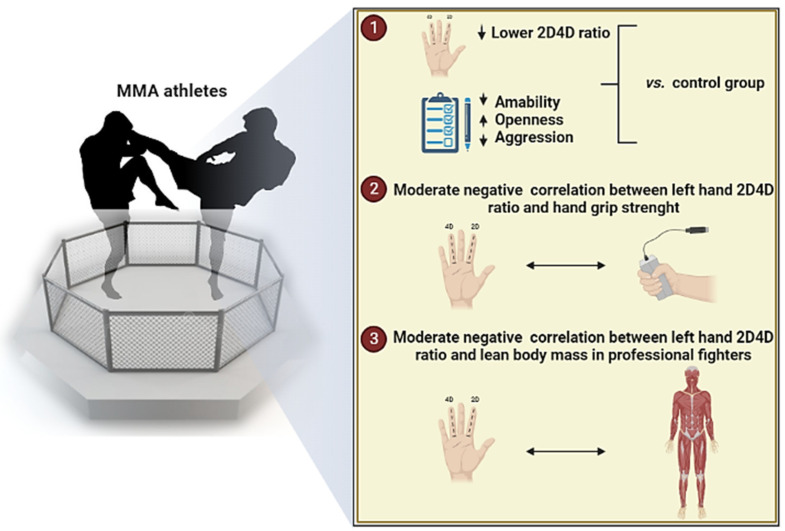
Overview of the main findings involved in MMA athletes.

The initial hypothesis of the present study has been partially confirmed, revealing that MMA athletes presented lower 2D:4D compared to the control group in both hands. In addition, we found a significantly moderate negative correlation between left hand 2D:4D and HGS in professional and amateur MMA athletes. Moreover, a negative correlation between left 2D:4D and LBM was observed in professional athletes. We also found differences concerning psychometric variables, i.e., lower BFI agreeableness and BPQ anger, and higher BFI openness scores in professional athletes than in controls (Figure 4). There was an overall positive correlation between BPQ anger and right and left 2D:4D in all subjects. However, we did not observe substantial associations between other psychometric variables or wins with 2D:4D. An overview of the main findings is shown in Figure 6. These promising findings suggest that 2D:4D evaluation in combination with other measures is a valuable tool for determining the potential athleticism of an individual. The possibility of applying the 2D:4D ratio as one of the choice criteria for predisposition—for direct physical fighting with an opponent—can be a useful measure in recruiting a team.

Although this was the first study investigating prenatal androgenization in MMA athletes, the lower 2D:4D in athletes than controls was expected, considering the evidence in other athletes, such as soccer players [7]. In addition, it was also reported that athletes with lower 2D:4D present better performance in sumo wrestling [13] and Greco–Roman wrestling [14], and that more androgenized men tend to exhibit higher preferences for practicing combat sports (i.e., wrestling, judo, boxing, and kickboxing) rather than aerobics or team sports [11,12]. Furthermore, it was observed that a lower 2D:4D is associated with a lower body fat percentage, greater bone mineral content, and greater bone mineral density, especially in men, which could plausibly be related to prenatal androgen exposure [38]. Thus, 2D:4D might be a biomarker of one’s fate.

Our results corroborate a study conducted by Krakowiak et al. [39]; they observed a negative correlation between the 2D:4D ratio and LBM in diverse sports modalities (football, volleyball, kayaking, rowing, and athletics). In particular, optimal LBM in MMA professional athletes may contribute toward improving health status and peak athletic performance, while decreasing the risk of injuries [40]. Furthermore, muscle mass indirectly helps enhance wrestling and grappling, improving one’s chances of knocking out the opponent while making it harder for the opponent to take one down. Although the link between the 2D:4D ratio and physical characteristics is still not fully clarified, androgens are considered beneficial for athletic performance by exerting positive effects on muscle tissue. Hence, the 2D:4D ratio potentially proves helpful in evaluating predisposition to practicing certain sports skills and disciplines.

One of the possible hypotheses as to why there is lower 2D:4D in athletes (according to the literature) is that prenatal androgenization may influence metabolic parameters, such as VO_2_max [9] and muscular strength [41]; this is because in utero male sexual differentiation and gonadal development are highly dependent on T release [18]. Consequently, higher levels of prenatal T may exert organizational effects on testicles and influence T release in adult life. Although a relationship between 2D:4D and HGS appears plausible for theoretical reasons, previous studies failed to detect associations between HGS and 2D:4D in healthy young men [42]. Interestingly, an important finding was that HGS may be negatively related to 2D:4D in the left hand of an MMA athlete. In the current study, we chose to perform the correlation test between HGS and prenatal androgenization in the athletes only, to avoid confounding factors, such as big differences in physical fitness. Despite [42] statistically controlling the analyses to engagement in (regular) physically demanding work (i.e., basketball-playing, and weightlifting), the modality specificity practiced and recreational aspects influence the frequency, intensity, and time of training, which may interfere with the HGS values.

Previous studies also failed to find associations between basal T and 2D:4D [5], but in a challenging condition (i.e., an aggressive video of rugby tackles), there was a highly significant increase in HGS and modest changes in T, physical aggression, and emotional stability in young men. These prior data indicate that low 2D:4D may be related to higher T release following an aggressive stimulus [5] and, therefore, it constitutes a predictive marker of free testosterone responsiveness when trained men are physically challenged, even though it is not associated with basal T values [6]. These relationships may in part explain the link between a low digit ratio and high performance in competitive sports. Furthermore, these findings are reinforced by a study that showed a low digit ratio predicting high physical aggression after a challenging situation of video aggression stimuli in young male non-athletes [43].

In our study, we found lower levels of BPQ anger in MMA athletes, which is in agreement with the view that combat sports may lower anger and help individuals to acquire emotional self-control [44]; however, we did not find differences in physical aggression. In addition, superficial analyses of our data (not shown) discarded the association between BPQ anger and HGS. Although martial arts and combat sports are often associated with aggressiveness by the general public, there is evidence of less verbal and physical manifested aggressiveness in combat sports (i.e., Judo) practitioners in comparison with team sports [45]. Thus, based on the outcomes found here and the investigations of other researchers, it can be stated that aggressive behavior can be altered, modified, relieved, or retarded through exercise training routines.

For this purpose, our study was designed with MMA athletes that performed aggressive maneuvers (with sparring) as training routines, which may have similar effects to the challenge conditions imposed by a previous study [5]. Concerning new data for the MMA population—we found lower BFI agreeableness and higher BFI openness in MMA athletes than in the controls. Considering the nature of this modality, where superiority over the rival is not shown on an object (e. g., a ball) or a distance (e.g., a marathon), but directly on the rival’s body (by potentially harmful techniques until knockout), it remains unclear if practicing and competing in MMA have led athletes to these types of behaviors or if men with natural lower agreeableness tend to choose MMA practicing. It is known that a higher level of openness to experience is characteristic of risky sports participants [46], which partially explains the higher levels of BFI openness in MMA athletes compared to non-athletes. BFI openness expresses exploratory behavior, which, mainly during competition, may be useful to explore a wide range of maneuvers, such as kicks, punches, holds, joint-locks, strangles, blows, and lunges, to surprise the opponent and be decisive in a fight challenge.

One may consider the psychometric and behavioral variables [20] discussed—that the relationship between physical fitness and low 2D:4D may be mediated through exercise frequency, suggesting that the organizational effects of prenatal T may predict a type of behavior that interferes with exercise frequency, and adequate periodization leads to increased strength and performance in male athletes. In our study population, all athletes reported up to 1:30 h/day training. However, we did not examine the relationship between 2D:4D and training frequency, as in Brazil, most MMA practitioners (whether professionals or amateurs) are not full-time athletes, which may impact recovery and training adaptations. From a practical perspective, the individual athlete’s psychology and perceptual responses to a stimulus is a complex and multi-faceted phenomenon, which might arise due to a variety of different mechanisms. Performance measures represent the most sport-specific outcomes but psychological measures are modulated by several genetic and epigenetic factors, which clarify our findings.

Our findings suggest that, within a similar physically active group of MMA athletes, the correlation we found between left 2D:4D and the HGS index may reflect T organizational effects. One essential aspect of the practical application of the present research is that prior 2D:4D, HGS, and psychological factor screening might provide efficient data for analyses and important feedback to determine key performance predictors in a particular competition, but this aspect does not explain full sports performances and it needs to be included with other fitness measures. The complexities and profound variabilities in muscle functioning highlight the need to better understand the multidimensional array of interacting factors that determine the trajectory of combat sports performances. Additionally, there should be further longitudinal studies with larger samples of athletes to confirm a relationship between 2D:4D in both hands and HGS in this specific population. Follow-up studies should be conducted to shed light on the behaviors of these athletes.

On the other hand, a significant result of the current study was that there was no significant association between prenatal androgenization and win outcome. Considering these findings, and as a possible explanation, we speculate that MMA sports performance is a complex and multi-faceted phenomenon, which might require a variety of demands, including technical, physical, biological, and sensorimotor skills. Consequently, there are a substantial number of techniques available to athletes and a broad variety of methods or strategies used to accomplish victories. In this sense, knowing multiple combinations of factors might determine a win at the highest level of MMA competition.

Some limitations of the present study should be highlighted. Our sample represents a few MMA gym experiences, limiting the generalizability of the results. The cross-sectional nature of this study prevents the ability to identify any causal relationship between study variables and outcomes. Although 2D:4D is recognized as a biomarker of prenatal T and participants with osteo fractures were excluded from the study (due to well-known risk factors from sports practice accidents, such as dislocating the phalanx), some residual confounders of measure could not be completely ruled out. Another important limitation is that testosterone levels were not assessed in this study. Although hormone measures are usually highly variable, future studies should address testosterone concentrations at both baseline and challenging conditions. Important outcomes, such as training experience, familiar income, and mental health disorders, were not evaluated. In future studies, the focus should be on researching distinct training levels and holistic assessment models of athletes.

## 5. Conclusions

In summary, MMA male athletes presented lower 2D:4D ratios in both hands than the control group. We observed that left hand 2D:4D was negatively correlated with HGS and LBM in professional athletes but it did not determine wins in MMA. Interestingly, these athletes presented significant differences in psychometric variables: lower BFI agreeableness, higher BFI openness, and lower BPQ anger than the control group. Beyond this, BPQ anger was also positively correlated with 2D:4D in both hands (in professional athletes and non-athletes). These findings suggest that including the 2D:4D measurement (in conjunction with other measures) is important in determining potential athleticism or talent in MMA.

## Figures and Tables

**Table 1:** Sample characterization and descriptive statistics.

	Athletes	Non-Athletes	*p* Value
Age (y) ^b^	25 (18–39) n = 69	22 (18–35) n = 53	0.002 *
Body Mass (Kg) ^a^	76.07 ± 13.85 n = 69	76.22 ± 11.12 n = 53	0.946
Heigh (cm) ^a^	176 ± 0.73 n = 69	175 ± 0.61 n = 53	0.664
BMI (kg/m^2^) ^a^	24.48 ± 3.63 n = 69	24.70 ± 2.69 n = 53	0.711

Note: ^a^ Parametric data: Results are described as mean ± standard deviation. ^b^ Non parametric data: Results are described as median (minimum–maximum). * *p* < 0.05.

**Table 2:** Association between prenatal androgenization and winning outcome.

	**Right High 2D:4D**	**Right Low 2D:4D**
High Wins	18 (26.47%)	15 (22.05%)
Low Wins	23 (33.82%)	12 (17.64%)
	**Left High 2D:4D**	**Left Low 2D:4D**
High Wins	13 (39.39%)	20 (60.61%)
Low Wins	16 (45.71%)	19 (54.29%)

Note: results are described as absolute frequency and relative frequency.

## Data Availability

The datasets used and/or analyzed in the current study will be made available from the corresponding author upon reasonable request.
